# Linking EEG markers of oscillopathy and default mode network dysfunction in Alzheimer’s disease

**DOI:** 10.3389/fnhum.2026.1750878

**Published:** 2026-06-05

**Authors:** Chaowen Chen, Dania Jose, Peter K.-H. Cheng, John J. Massa, Wen Li

**Affiliations:** Louis A. Faillace, MD, Department of Psychiatry and Behavioral Sciences, University of Texas Health Science Center, Houston, TX, United States

**Keywords:** Alzheimer’s disease, artificial intelligence, default mode network, disconnection, EEG markers, oscillopathy, transcranial stimulation

## Abstract

Alzheimer’s disease (AD) is a progressive neurodegenerative disorder characterized by cognitive decline and multifaceted neuropathology. Although fluid biomarkers cerebrospinal fluid (CSF) amyloid-*β* and tau and imaging biomarkers positron emission tomography (PET) have substantially advanced AD diagnostics, electroencephalography (EEG) remains a comparatively underutilized tool despite its unique potential. EEG biomarkers capture oscillatory abnormalities (oscillopathies), providing a dynamic window into network-level dysfunction in AD pathophysiology that is not accessible through static molecular or structural measures. Of particular interest are EEG indices linked to dysfunction in the default mode network (DMN), a large-scale intrinsic network increasingly recognized as an epicenter of AD vulnerability. This review synthesizes evidence for key EEG oscillopathy markers in AD—including spectral slowing, gamma deficits, and reduced long-range synchrony—and maps these alterations onto DMN dysfunction within a hierarchical cascade spanning molecular pathology to cognitive impairment. We further explore how frequency-specific transcranial alternating current stimulation (tACS) may both mechanistically interrogate and therapeutically target the oscillopathy-DMN link, drawing on causal evidence from combined EEG-fMRI studies and emerging clinical trials. Finally, we discuss how artificial intelligence (AI), applied to large-scale multimodal datasets, may transform oscillopathy markers into tools for personalized diagnosis, prognosis, and closed-loop neuromodulation. By integrating EEG, network neuroscience, and targeted stimulation, this narrative review positions oscillopathy as a central and clinically accessible feature of AD-related network dysfunction.

## 1 Introduction

Alzheimer’s disease (AD) is the most common cause of dementia, affecting an estimated 6.9 million Americans aged 65 and older and tens of millions globally. As the population ages, prevalence is projected to reach 13.8 million by 2060 (Alzheimer's Association, 2024). Risk doubles approximately every five years after age 65, and by 2030, 20% of the U.S. population will be 65 or older (Vespa et al., 2020). AD imposes profound personal, societal, and economic burdens, creating an urgent need to deepen our understanding of its pathology and to develop effective tools for early detection, monitoring and treatment.

### 1.1 Current hypotheses of AD pathology

Akin to its global functional impacts, AD is multifactorial in pathogenesis and progression. Converging mechanistic hypotheses suggest that interacting molecular, cellular, and vascular processes drive a multiscale cascade, linking synaptic dysfunction to large-scale network disintegration and ultimately to cognitive decline.

#### 1.1.1 The amyloid cascade hypothesis

The “amyloid cascade hypothesis” remains a central model of AD pathogenesis, proposing that the initial accumulation of amyloid-*β* (Aβ) plaques triggers a downstream cascade leading to the formation of neurofibrillary tangles composed of hyperphosphorylated tau protein (Musiek and Holtzman, 2015). Tau, a microtubule-associated protein responsible for cytoskeletal stabilization, becomes hyperphosphorylated and dysfunctional in AD, forming misfolded aggregates associated with neuronal degeneration (Braak and Braak, 1991). However, the amyloid cascade may not be the exclusive driver of disease progression. In sporadic AD, tau pathology can emerge in certain regions before widespread cortical amyloid deposition, initially appearing in structures such as the locus coeruleus and subsequently spreading in a predictable pattern to the transentorhinal cortex, hippocampus, and neocortex (Braak and Del Tredici, 2014, 2015). Early tau pathology correlates more closely with neuronal dysfunction and often precedes overt cognitive decline. Furthermore, FDA-approved anti-amyloid monoclonal antibodies have demonstrated robust plaque reduction but only modest clinical benefits, suggesting that amyloid represents one of several interacting pathological processes rather than the exclusive determinant of disease progression (Heidebrink and Paulson, 2024).

#### 1.1.2 The neurovascular hypothesis

Beyond protein aggregation models, the *“neurovascular hypothesis”* proposes that vascular dysfunction plays a critical role in AD pathogenesis. Early in the disease, regions such as the posterior cingulate cortex (PCC), precuneus, and hippocampus exhibit hypoperfusion, potentially leading to energy deficits and oxidative stress that contribute to neuronal dysfunction (Love and Miners, 2016). Moreover, in AD, neuronal activity may no longer elicit appropriate local blood flow increases—a phenomenon termed “neurovascular uncoupling.” This dysregulation likely stems from endothelial dysfunction and loss of astrocytes and pericytes (Tarantini et al., 2017). Additionally, the blood–brain barrier (BBB) becomes leaky in early AD, particularly in the hippocampus, permitting entry of neurotoxic proteins and inflammatory cells while impairing amyloid-*β* clearance (Montagne et al., 2020)

#### 1.1.3 The disconnection hypothesis: a network perspective

More recently, the *“disconnection hypothesis”* conceptualizes AD as a progressive disconnection syndrome (Briels et al., 2020a; Brier et al., 2014; Delbeuck et al., 2003). This framework posits that amyloid, tau, and vascular pathologies progressively dismantle the brain’s finely tuned intra- and inter-regional neuronal communication networks. Rather than viewing AD as merely a proteinopathy or vasculopathy, the disconnection hypothesis emphasizes that cognitive decline emerges from the breakdown of coordinated large-scale neural network activity.

Among these networks, dysfunction of the default mode network (DMN)—a principal intrinsic large-scale network (Margulies et al., 2016)—is strongly implicated in AD. The DMN supports core cognitive functions (Raichle, 2015) such that its dysfunction provides a direct pathway to the profound cognitive decline observed in AD, rendering it particularly vulnerable to pathological disruption. Critically, the disconnection hypothesis bridges levels of analysis: molecular and vascular pathologies do not merely damage neurons—they impair the synchronized communication required for network integrity and cognitive function.

### 1.2 The current landscape of AD biomarkers

Current AD biomarkers are dominated by fluid and imaging markers. These markers primarily index molecular pathology and are relatively insufficient at measuring large-scale network dysfunction.

#### 1.2.1 Fluid biomarkers

Cerebrospinal fluid (CSF) biomarkers are widely used in AD diagnosis and staging, primarily grounded in the amyloid cascade model. Early-stage AD is characterized by reduced CSF Aβ_42_, attributed to its aggregation into brain plaques (Alawode et al., 2022; Xiong et al., 2021). In contrast, CSF total-tau (t-tau) and phosphorylated tau (p-tau181) levels increase after amyloid deposition, indicating early neuronal injury and tau pathology—often preceding widespread neurofibrillary tangle formation (Mattsson-Carlgren et al., 2022). Distinct CSF profiles strongly correlate with AD pathology, including (1) decreased Aβ_42_ and Aβ_42_/Aβ_40_ ratio, (2) elevated t-tau and t-tau/ Aβ_42_ ratio, and (3) elevated p-tau181 and p-tau181/ Aβ_42_ ratio (Karch and Goate, 2015; Li et al., 2020). Recent advances have enabled blood-based biomarkers, such as an elevated plasma Aβ_42_/Aβ_40_ ratio, offering a less invasive and increasingly accessible diagnostic approach (Musiek et al., 2018). While these fluid markers provide critical insight into molecular pathology and disease staging, they do not directly capture the dynamic network-level dysfunction that underlies cognitive impairment.

#### 1.2.2 Imaging biomarkers

Positron emission tomography (PET) imaging—particularly with amyloid and tau tracers—has become increasingly integrated into clinical practice for AD diagnosis and staging. Its utility is supported by strong regional concordance between premortem PET findings and postmortem neuropathological confirmation (Clark et al., 2012; Leuzy et al., 2022). Magnetic resonance imaging (MRI) provides complementary structural and functional markers. Structural MRI detects patterns of regional atrophy, particularly in medial temporal and posterior cortical regions, while functional MRI (fMRI) measures blood-oxygen-level-dependent (BOLD) signals, to assess intrinsic brain activity and inter-regional connectivity associated with neuropsychiatric functions and intrinsic inter-regional connectivity (Kwong et al., 1992; Raichle et al., 2001). Notably, resting-state fMRI has consistently demonstrated disrupted connectivity within large-scale networks—especially the default mode network—in AD (Badhwar et al., 2017; Sorg et al., 2007). While imaging biomarkers offer critical spatial information regarding molecular burden and network integrity, they remain indirect proxies of neural activity and lack the temporal resolution needed to capture rapid oscillatory dynamics underlying network communication.

#### 1.2.3 Limitations of current biomarkers

Despite their diagnostic value, current AD biomarkers have significant limitations. First, invasiveness and risk remain concerns; lumbar puncture is invasive, and PET imaging involves radiation exposure. Second, cost and accessibility constrain widespread implementation, as imaging and validated biomarker assays remain expensive and are not uniformly available across healthcare systems. Third, and more fundamentally, fluid and imaging biomarkers primarily index molecular burden and structural change, providing static snapshots of pathology rather than real-time measures of neural communication. Although molecular alteration may precede clinical symptoms by decades, these markers do not directly capture the dynamic network dysfunction that more closely parallels cognitive decline. Taken together, these limitations highlight the need for biomarkers that capture real-time network dysfunction rather than static pathology burden. Electroencephalography (EEG), with its millisecond temporal resolution and direct sensitivity to neural synchrony, offers precisely such a window into network-level dysfunction.

### 1.3 Electroencephalography (EEG) markers

Rapid advances in EEG analysis and neurocomputational methods have identified promising EEG indices of AD-related network dysfunction (Jiang et al., 2024; Zheng et al., 2023). With millisecond temporal resolution and high test–retest reliability, EEG offers a direct and dynamic measure of neural synchrony—an essential mechanism underlying large-scale network communication (Buzsaki and Draguhn, 2004; Etkin and Mathalon, 2024; McMackin et al., 2019). Beyond its temporal precision, EEG is portable, cost-effective, and scalable, making it well-suited for longitudinal monitoring and translational application. While fluid and imaging markers remain gold-standard tools for diagnosing and staging AD, EEG is increasingly recognized for its potential to characterize dynamic network-level dysfunction that more closely parallels cognitive decline. When combined with large-scale AI-driven analytic approaches, EEG biomarkers offer promising measurements for disease detection, diagnosis, longitudinal monitoring, and evaluation of therapeutic response.

Beyond prior reviews of EEG abnormalities in AD (Jiang et al., 2024; Zheng et al., 2023), this narrative review advances the framework that EEG oscillopathy markers, interpreted within a network context, may serve as clinically accessible indicators of DMN vulnerability and large-scale communication failure. Building on this perspective, we examine the convergence of large-scale EEG data, AI-based modeling, and frequency-targeted non-invasive brain stimulation (NIBS)—particularly transcranial alternating current stimulation (tACS)—positioning oscillatory biomarkers at the center of a network-informed paradigm for AD monitoring and intervention. In the following sections, we (1) synthesize evidence on key EEG oscillopathy markers in AD; (2) delineate the mechanistic link between oscillatory abnormalities and DMN dysfunction; (3) review emerging tACS studies that causally probe and modulate these oscillatory disturbances; and (4) explore AI-driven approaches to advance precision monitoring and personalized neuromodulation strategies.

## 2 EEG oscillopathy—the electrophysiological signature of AD

Within the disconnection framework of AD, understanding disease progression requires insights into large-scale communication breakdown. Network function depends on precisely coordinated neural oscillations, which synchronize activity within and across distributed brain regions (i.e., neural synchrony) (Buzsaki and Draguhn, 2004; Fries, 2005). These neural oscillations manifest as rhythmic patterns of electrical activity generated through interactions between cortical and subcortical circuits; they are conventionally categorized into frequency bands: delta (0.5–4 Hz), theta (4–7 Hz), alpha (8–12 Hz), beta (12–30 Hz), and gamma (30–80 Hz) (Buzsaki and Draguhn, 2004). Oscillopathy refers to the pathological disruption of these rhythmic processes at local (microcircuit) or large-scale (network) levels and has been implicated across multiple neuropsychiatric conditions (Akiki et al., 2017; Greicius et al., 2007; Llinas et al., 1999; Whitfield-Gabrieli et al., 2009). In AD, accumulating evidence indicates that oscillatory abnormalities are not incidental findings but represent core features of network dysfunction. Resting-state EEG (rsEEG) provides a non-invasive method to quantify intrinsic oscillatory activity. By capturing deviations in oscillatory power, synchrony, and cross-frequency interactions, EEG biomarkers offer a direct window into large-scale communication failure in AD. Importantly, these oscillatory alterations should not be viewed merely as downstream epiphenomena of neurodegeneration. Rather, they may reflect system-level processes through which molecular and cellular pathologies translate into network-level disintegration and cognitive impairment.

### 2.1 Core oscillopathy markers: a functional perspective

#### 2.1.1 Frequency bands and their functional relevance

Neural oscillations across canonical frequency bands subserve distinct yet interdependent cognitive processes. Theta rhythms are linked to memory encoding and retrieval (Klimesch, 1999); alpha oscillations contribute to attentional modulation and inhibitory control (Jensen and Mazaheri, 2010); beta activity supports sensorimotor integration and higher-order cognitive processing (Engel and Fries, 2010); and gamma oscillations are associated with perceptual experience and conscious awareness (Lutz et al., 2004). Importantly, these rhythms do not operate in isolation. Their spatial distribution and temporal coordination—both locally and across long-range networks—enable the integration of distributed neural processes into coherent cognitive functions (Engel et al., 2001). Disruption within or between these frequency bands, therefore, has the potential to destabilize large-scale network communication, rendering oscillatory abnormalities particularly relevant to the network disconnection observed in AD.

### 2.2 Spectral slowing (the alpha/theta shift)

One of the most robust electrophysiological signatures of AD is EEG slowing, characterized by increased power in slow frequencies (delta, theta), decreased power in faster frequencies (alpha, beta, gamma), and elevated slow-to-fast frequency power ratios (e.g., theta/alpha, theta/beta).

#### 2.2.1 Increased power in slower frequencies

Numerous studies report significantly elevated theta power in AD patients compared to healthy controls (HCs). Babiloni et al. (2020) reported increased global delta and theta power in resting state-EEG from 100 AD patients relative to 100 HCs. Using exact low-resolution brain electromagnetic tomography (eLORETA; Babiloni et al., 2020; Pascual-Marqui, 2007; Pascual-Marqui et al., 1994), Musaeus et al. (2018) similarly demonstrated elevated global theta power in 117 AD and 117 mild cognitive impairment (MCI) patients compared with 138 HCs, with the increase being most pronounced in AD. Relative theta power—calculated as absolute theta power divided by total power across 1–45 Hz—achieved 72.9% classification accuracy in distinguishing AD from HC (Musaeus et al., 2018). Additionally, increased global theta and delta power have been associated with decreased CSF Aβ_42_, whereas reductions in alpha and beta power correlate with elevated CSF p-tau and t-tau, further linking oscillatory slowing to core AD pathology (Smailovic et al., 2018).

#### 2.2.2 Decreased power in faster frequencies

Reduced alpha and beta power is consistently observed in AD (Benwell et al., 2020; Huang et al., 2000; Moretti et al., 2004; Roh et al., 2011). These reductions appear particularly prominent in posterior cortical regions (Babiloni et al., 2013; Jovicich et al., 2019), and decreased posterior alpha power correlates with hippocampal atrophy (Babiloni et al., 2009). Lower global alpha and beta power is also associated with elevated CSF p-tau and t-tau, suggesting a linkage to advanced neuronal injury (Cecchetti et al., 2021; Smailovic et al., 2018). Notably, CSF p-tau levels have been directly associated with slowing of alpha peak frequency (Kramberger et al., 2013). Gamma-band power is likewise reduced in AD, particularly within frontal cortices (Casula et al., 2022).

#### 2.2.3 Increased slow/fast frequency power ratios

Composite metrics such as the theta/alpha ratio further highlight EEG slowing and may enhance diagnostic accuracy. The ratio of high-frequency (alpha + beta) to low-frequency (delta + theta) power distinguished 35 AD patients from 35 HCs (Bennys et al., 2001). More recently, a frontal alpha/theta ratio effectively distinguished 47 early-onset AD patients from 49 HCs, with an area under the curve (AUC) of 0.85, and sensitivity and specificity above 80% (Ozbek et al., 2021).

### 2.3 High-frequency disruptions (gamma band abnormalities)

Gamma oscillations provide a critical window into local circuit dysfunction in AD, characterized by reduced gamma power. Impaired stimulus-induced gamma synchrony, particularly within medial temporal lobe (MTL) regions, suggests compromised engagement of fast oscillatory activity during cognitive processing in AD patients (Casula et al., 2022; Koenig et al., 2005; van der Hiele et al., 2007; van Deursen et al., 2008).

Converging evidence from animal models provides a mechanistic link for these findings. Tau pathology has been linked to dysfunction of parvalbumin-positive (PV+) interneuron dysfunction and gamma deficits: Verret et al. (2012) demonstrated that impaired PV+ interneuron function leads to reduced gamma power and memory deficits in mouse models. More recently, 40-Hz sensory stimulation was shown to reduce amyloid and tau pathology in mouse AD models, potentially through enhancement of gamma oscillatory activity and recruitment of PV+ interneurons (Adaikkan et al., 2019; Martorell et al., 2019).

### 2.4 Disrupted functional connectivity (loss of synchrony)

Beyond spectral power alterations, functional connectivity measures are essential for mapping large-scale network disintegration in AD. Different metrics capture distinct coupling mechanisms: phase-based metrics index oscillatory synchronization, coherence-based measures reflect frequency-specific linear coupling, and directed measures assess the directionality/causality of information flow (see Table 1 for specific functions). Given emerging evidence linking oscillopathy and DMN dysconnectivity in AD, employing complementary connectivity measures enables a more physiologically grounded characterization of network pathology. Lag-based and directed measures are particularly valuable in limiting spurious connectivity driven by volume conduction (Bastos and Schoffelen, 2016). Moreover, this multidimensional feature space provides a rich substrate for advanced analytical approaches, including AI-based modeling aimed at detecting subtle and distributed pathological signatures.

**Table 1:** rsEEG markers of AD.

rsEEG biomarkers	Measurement	Changes in AD
Power	Magnitude of EEG oscillations within a frequency band	↑ theta and delta bands ↓ alpha and beta bands (esp. posterior regions)
Peak frequency	Frequency with maximal power of a given oscillation	↓ alpha peak frequency
Connectivity (un-directed)		
Phase-locking value (PLV)	Phase synchronization between oscillatory signals (temporal coordination)	↓ theta (frontal), alpha, & beta bands
Phase-amplitude coupling (PAC)	Cross-frequency interactions (hierarchical coordination between slow and fast oscillations)	↓ theta–gamma
Global field synchronization (GFS)	A global measure of large-scale phase alignment (network-level synchrony)	↓ lower alpha, beta, & gamma bands
Spectral coherence	Frequency-specific linear coupling (shared oscillatory power across regions)	↓ alpha band
Lagged phase synchronization (LPS)	Phase synchronization after removing zero-lag volume conduction	↓ delta band (most regions) ↓ theta band (right DLPFC-PIP)
Connectivity (directed)		
Granger causality/directed transfer function	Directional information flow (network causality)	↓ posterior-to-anterior projection (esp. alpha band)

DLPFC, dorsolateral prefrontal cortex; PIP, posterior inferior parietal lobule.

#### 2.4.1 Decreased phase-locking value

Phase-locking value (PLV) quantifies the consistency of phase difference between oscillatory signals from two EEG channels over time within a specific frequency band (Lachaux et al., 1999). High PLV indicates stable phase relationships and synchronized neural communication, whereas low PLV reflects disrupted connectivity (Kirschner et al., 2012). Reduced rsEEG PLV in theta band, particularly within frontal regions, has been reported in 26 AD patients relative to 29 HCs (Wang et al., 2025). Briels et al. (2020b) further demonstrated reproducible reductions in alpha and beta PLV within two large AD cohorts (*N* = 83 and *N* = 80) versus HCs, suggesting widespread disruption of oscillatory synchronization across frequency bands.

#### 2.4.2 Decreased spectral coherence

Spectral coherence indexes synchronization between two EEG signals at a specific frequency over time, reflecting both phase consistency and amplitude correlation across regions (Jelic et al., 2000). In contrast to phase-amplitude coupling (PAC), which captures cross-frequency interactions, spectral coherence specifically assesses within-frequency synchrony (e.g., how consistently two regions oscillate together at alpha frequency) (Bastos and Schoffelen, 2016; Zhang et al., 2014). In AD, spectral coherence is frequently reduced, reflecting network disconnection. A meta-analysis by Fischer et al. (2023) reported significant reductions in alpha-band coherence in 24 of 34 studies, particularly in long-range frontoparietal and frontotemporal connections.

#### 2.4.3 Reduced lagged phase synchronization

Lagged phase synchronization (LPS) quantifies non-instantaneous phase relationships between EEG signals, while excluding zero-lag coupling. Reduced LPS has been observed in AD, indicating weakened inter-regional synchrony. Hata et al. (2016) reported markedly decreased delta-band LPS across multiple cortical regions in AD relative to HCs. Delta rhythms are involved in large-scale integrative processes (Bruns and Eckhorn, 2004). Although delta activity may increase as a compensatory response in neurodegeneration (Pathak et al., 2022), reduced lagged synchronization suggests progressive impairment of large-scale integrative processes (Gaubert et al., 2019).

#### 2.4.4 Global field synchronization

Global field synchronization (GFS) provides a reference-independent measure of phase synchronization across all EEG electrodes within a given frequency band (Koenig et al., 2005; Ma et al., 2014). Reduced GFS has been observed in lower alpha, beta, and gamma bands in AD (Koenig et al., 2005; Park et al., 2008), reflecting widespread desynchronization of large-scale networks. Importantly, GFS alterations correlate with clinical measures including MMSE, MoCA, and CDR scores in AD (Ma et al., 2014; Park et al., 2008). Furthermore, GFS decreases in higher-frequency bands correlate with elevated CSF p-tau and t-tau, linking these EEG anomalies to AD pathology (Smailovic et al., 2018).

### 2.5 Reduced effective connectivity

Beyond undirected synchrony measures, recent EEG studies have identified effective connectivity metrics to characterize directional interactions across brain networks. These approaches provide mechanistic insights into the integrity of information flow and hierarchical organization in AD. Several studies report reduced posterior-to-anterior phase transfer entropy gradient in alpha (Babiloni et al., 2004), beta (Dauwan et al., 2016), and broadband oscillatory activity (Blinowska et al., 2017) in AD, suggesting impaired forward propagation of neural signals. Similarly, posterior-to-anterior Granger causality (GC) in theta, beta, and gamma bands has been reported disrupted in AD (Juan-Cruz et al., 2016). These findings are consistent with MEG evidence demonstrating reduced resting-state information flow from posterior regions, including precuneus and visual cortex toward frontal and subcortical areas in AD (Engels et al., 2017), further supporting a breakdown of directed network communication.

### 2.6 Altered phase-amplitude coupling

Phase-amplitude coupling (PAC) describes the interaction whereby the phase of slower oscillation (delta or theta) modulates the amplitude of faster oscillation (beta or gamma). Slow rhythms are typically generated by large-scale networks such as thalamo-cortical circuits and regulate the timing of neuronal excitability (Qin et al., 2021). In contrast, faster oscillations reflect local cortical processing and fluctuate in amplitude according to the phase of slower rhythms (Hyafil et al., 2015). Through this cross-frequency interaction, PAC supports hierarchical organization in brain rhythms and coordinates local and global processing underlying cognitive functions such as memory and attention (Koster et al., 2018; Szczepanski et al., 2014). Disruption of PAC in AD may therefore reflect impaired dynamic integration across spatial and temporal scales. Indeed, Goodman et al. (2018) reported significantly reduced theta-gamma PAC in frontal and parietal regions in AD patients during a working memory (2-back) task compared with HCs.

### 2.7 Summary and limitations

In summary, EEG markers reveal significant oscillopathies across multiple dimensions, including spectral power, synchrony, directional connectivity, and cross-frequency coupling (Table 1). These anomalies have been linked to amyloid and tau pathology as well as large-scale network disruptions. Given the central role of oscillatory coordination in enabling inter-regional communication (Buzsaki and Draguhn, 2004; Tagliazucchi et al., 2012; Uhlhaas et al., 2009), these alterations further support conceptualizing AD as a disconnection syndrome (Brier et al., 2014; Delbeuck et al., 2003), involving not only neuronal degeneration but also impaired network-level integration.

Despite their promise, the literature on EEG markers has important limitations. First, publication bias and selective reporting may inflate apparent consistency in literature; some studies report attenuated or non-significant effects, particularly in early or prodromal stages (Cassani et al., 2018), or when alternative analytic pipelines are applied (Badhwar et al., 2017). Sensitivity can also depend on clinical stratification and recording contexts (Jelic et al., 2000). Second, pharmacological treatment may confound oscillatory signatures. Acetylcholinesterase inhibitors, commonly used in AD, can systematically alter oscillatory activity—typically reducing theta and delta power while exerting variable effects on alpha power depending on the specific agent (e.g., donepezil vs. rivastigmine; Babiloni et al., 2013). Third, EEG measures are inherently state-dependent. Variations in alertness, cognitive engagement, and task demand can substantially influence oscillatory power and synchrony, complicating the differentiation between stable disease biomarkers from transient physiological fluctuations. Finally, methodological heterogeneity including differences in preprocessing, epoch selection, and electrode configurations—limits reproducibility and challenges the establishment of standardized diagnostic thresholds. Nevertheless, advances in computational modeling, including AI-based approaches capable of capturing multidimensional and state-sensitive patterns, may help address these limitations.

## 3 The convergent oscillation-DMN pathology

### 3.1 The default mode network: function and vulnerability

The Default Mode Network (DMN), encompassing core hubs such as the posterior cingulate cortex (PCC), medial prefrontal cortex (mPFC), and medial temporal lobe (MTL) structures, subserves fundamental cognitive processes including episodic memory retrieval, self-referential thought, future simulation, and mind-wandering (Raichle, 2015). Critically, the DMN exhibits anti-correlated dynamics with task-positive networks, typically deactivating during externally oriented cognitive demands to reduce interference and optimize performance (Fox et al., 2005). Given its central role in autobiographical memory and higher-order cognition, disruption of DMN integrity contributes to the characteristic cognitive decline observed in Alzheimer’s disease (AD).

The selective vulnerability of the DMN in AD likely reflects a convergence of metabolic, molecular, and vascular factors: (1) *High baseline metabolic activity*—DMN hubs exhibit persistently elevated resting-state glucose metabolism and synaptic activity, rendering them susceptible to metabolic stress, mitochondrial dysfunction, and oxidative damage (Buckner et al., 2005); (2) *Early amyloid deposition*—Amyloid-PET studies consistently demonstrate that fibrillar amyloid-*β* accumulates early and prominently within DMN hubs, particularly the PCC and precuneus, spatially overlapping with intrinsic connectivity architecture (Palmqvist et al., 2017); (3) *Early tau accumulation*—MTL hubs of DMN (hippocampus, entorhinal cortex) are among the earliest regions to accumulate tau pathology, consistent with Braak staging that begins in the transentorhinal cortex and subsequently spreads along connected networks (Braak and Braak, 1991; Braak and Del Tredici, 2015); and (4) *Neurovascular vulnerability*—early hypoperfusion in the PCC, precuneus, and hippocampus may lead to energy deficits, impaired clearance mechanisms, and downstream neuronal dysfunction (Love and Miners, 2016).

### 3.2 Linking oscillopathy to DMN dysfunction

The intricate interplay of molecular (amyloid and tau), cellular (synaptic loss, interneuron dysfunction), and vascular (hypoperfusion, neurovascular uncoupling) pathologies in AD progressively destabilizes coordinated neural communication. Beyond structural degeneration, these processes perturb the electrophysiological architecture that sustains large-scale network integration. Here we map specific oscillopathy signatures onto DMN dysfunction, providing a mechanistic framework linking oscillatory disruptions to large-scale networks.

#### 3.2.1 Spectral slowing and loss of alpha synchrony mediate DMN dysfunction

##### 3.2.1.1 Alpha oscillations and DMN: an intrinsic relationship

Alpha oscillations, the dominant rhythm of intrinsic neural synchrony (Klimesch et al., 2007; Palva and Palva, 2007), are intrinsically linked to DMN function (Tagliazucchi et al., 2012; Tang et al., 2017). Simultaneous EEG-fMRI studies consistently demonstrate positive coupling between resting-state alpha power and DMN activity (Clancy et al., 2022; Jann et al., 2009; Scheeringa et al., 2012). Specifically, fMRI and source-localized EEG/MEG analyses further reveal that alpha-band synchrony functionally links posterior and anterior DMN hubs—particularly the PCC and mPFC—supporting large-scale intrinsic integration (Clancy et al., 2022; Coito et al., 2019; Hillebrand et al., 2016; Samogin et al., 2019). Functionally, both alpha oscillations and DMN contribute to internally oriented processing and disengagement from external sensory input during rest (Foxe and Snyder, 2011; Raichle and Snyder, 2007). Thus, alpha-band synchrony binds DMN hubs into a temporally coherent network and facilitates appropriate task-related deactivation when externally directed demands rise. In AD, this intrinsic alpha-DMN coupling becomes disrupted along two interrelated dimensions: (1) *spectral slowing*, characterized by reduced peak alpha frequency and increased lower-frequency power; and (2) *diminished alpha synchrony* across distributed cortical regions. Together, these alterations destabilize DMN coordination, contributing to both aberrant task-related hyperactivity and reduced resting-state functional connectivity.

##### 3.2.1.2 Spectral slowing mediates failed deactivation

When posterior alpha activity is attenuated and replaced by slower theta/delta oscillations in posterior DMN hubs (PCC, precuneus), the network’s capacity for dynamic modulation is compromised. Alpha oscillations normally support inhibitory gating and coordinated disengagement during externally directed tasks; their reduction weakens this regulatory mechanism. In fMRI, this disruption manifests as well-documented “failure to deactivate” DMN during cognitive tasks (Greicius et al., 2004; Lustig et al., 2003). Instead of appropriately downregulating activity, DMN hubs remain persistently active, interfering with task-positive network recruitment. This aberrant persistence of intrinsic activity has been associated with impaired attentional control and episodic memory performance in AD (Anticevic et al., 2012), suggesting that spectral slowing contributes to dysfunctional network competition and reduced cognitive efficiency.

##### 3.2.1.3 Loss of alpha synchrony mediates functional disconnection

Simultaneously, reduced long-range alpha-band coherence between core DMN hubs (e.g., PCC-mPFC, PCC-MTL) reflects breakdown of an inter-regional communication. Alpha-band synchrony facilitates large-scale information integration by establishing temporally coordinated windows for effective neuronal communication (Palva and Palva, 2007; Varela et al., 2001). When distributed DMN hubs fail to synchronize in the alpha range, coordinated network integration deteriorates, promoting functional fragmentation.

Consistent with this framework, reproducible reductions in alpha-band phase locking (PLV) during rsEEG have been reported in AD (Briels et al., 2020a), linking oscillatory desynchronization to disrupted large-scale coupling. Moreover, attenuation of the posterior-to-anterior phase transfer entropy gradient in the alpha band (Babiloni et al., 2004; Dauwan et al., 2016) suggests that directional information flow within DMN is selectively compromised. Together, these oscillatory abnormalities likely constitute the electrophysiological substrate underlying reduced DMN functional connectivity observed in resting-state fMRI studies of AD (Badhwar et al., 2017; Dennis and Thompson, 2014; Sheline and Raichle, 2013).

#### 3.2.2 Gamma disruption mediates local MTL dysfunction

Gamma oscillations critically depend on fast-spiking parvalbumin-positive (PV+) inhibitory interneurons, which generate precisely timed inhibitory postsynaptic potentials, that synchronize pyramidal cell firing at gamma frequencies (Cardin et al., 2009; Sohal et al., 2009). Reduced gamma power and impaired stimulus-induced gamma synchrony within MTL regions likely reflect local circuit pathology in key DMN hubs, thereby contributing to both DMN dysfunction and broader network instability. In AD, reductions in spontaneous gamma power and stimulus-induced gamma synchrony within MTL regions likely reflect interneuron dysfunction and local circuit disorganization within a core DMN hub (Koenig et al., 2005; Palop and Mucke, 2016; Verret et al., 2012). This gamma impairment may weaken microcircuit integrity in the hippocampus and entorhinal cortex, contributing to memory deficits and broader instability of the DMN (Casula et al., 2022; Martorell et al., 2019).

### 3.3 The cascade: from molecules to networks to cognition

**Figure 1:**
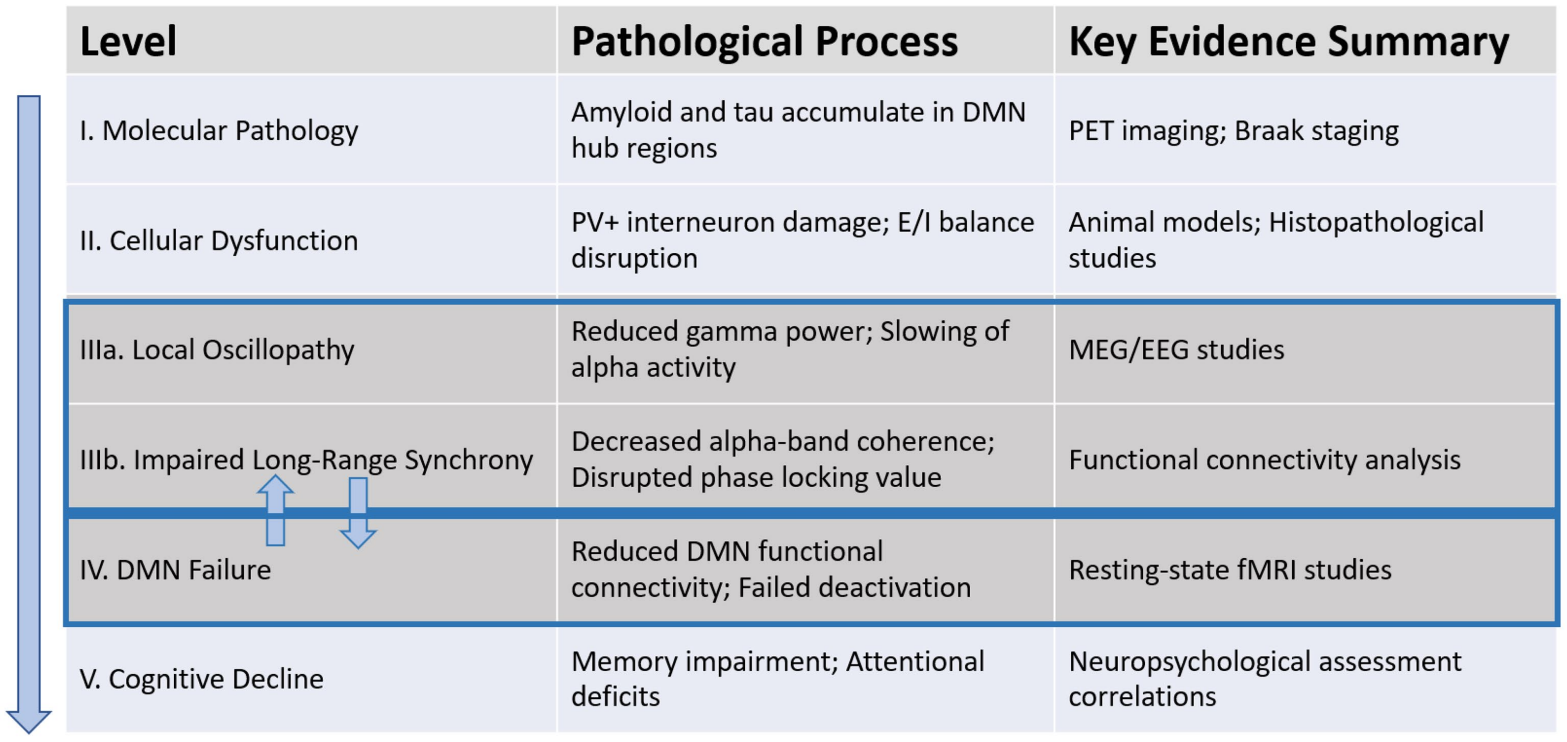
Oscillatory and DMN dysfunction in a multi-level pathological cascade model of Alzheimer’s disease. The progression from molecular pathology to clinical symptoms is depicted across five sequential levels, with Level III encompassing two interrelated sublevels of oscillopathy and mutual influence between Levels III and IV. Level I describes the accumulation of amyloid-beta and tau aggregates within default mode network (DMN) hubs. Level II illustrates subsequent cellular dysfunction, including damage to parvalbumin-positive (PV+) interneurons and disruption of excitation/inhibition (E/I) balance. Level III (Oscillopathy) is divided into two interacting components: Level IIIa represents local oscillatory abnormalities (reduced gamma power, alpha slowing), while Level IIIb reflects impaired long-range synchronization (decreased coherence, disrupted phase locking). These two sublevels exert bidirectional influence over one another, forming a feedback loop that amplifies network dysfunction. Level IV represents large-scale DMN failure, characterized by reduced functional connectivity and impaired task-induced deactivation. Critically, mutual influence exists between Levels III and IV: oscillatory disruptions both drive and are exacerbated by DMN network failure. Level V culminates in measurable cognitive decline, including memory impairment and attentional deficits.

Synthesizing the evidence presented above, we propose a hierarchical cascade spanning five progressive levels—from molecular pathology within DMN vulnerable regions to overt clinical symptoms (Figure 1). Within this framework, oscillopathy and DMN network failure represent critical systems-level intermediaries that bridge microscopic anomalies (Levels I–II) and clinical cognitive impairment in AD (Level V). Oscillopathy is not merely an epiphenomenon of network degeneration; rather, it constitutes a mechanistic conduit through which molecular and cellular abnormalities propagate into DMN dysfunction.

## 4 Targeting the link via tACS

**Figure 2:**
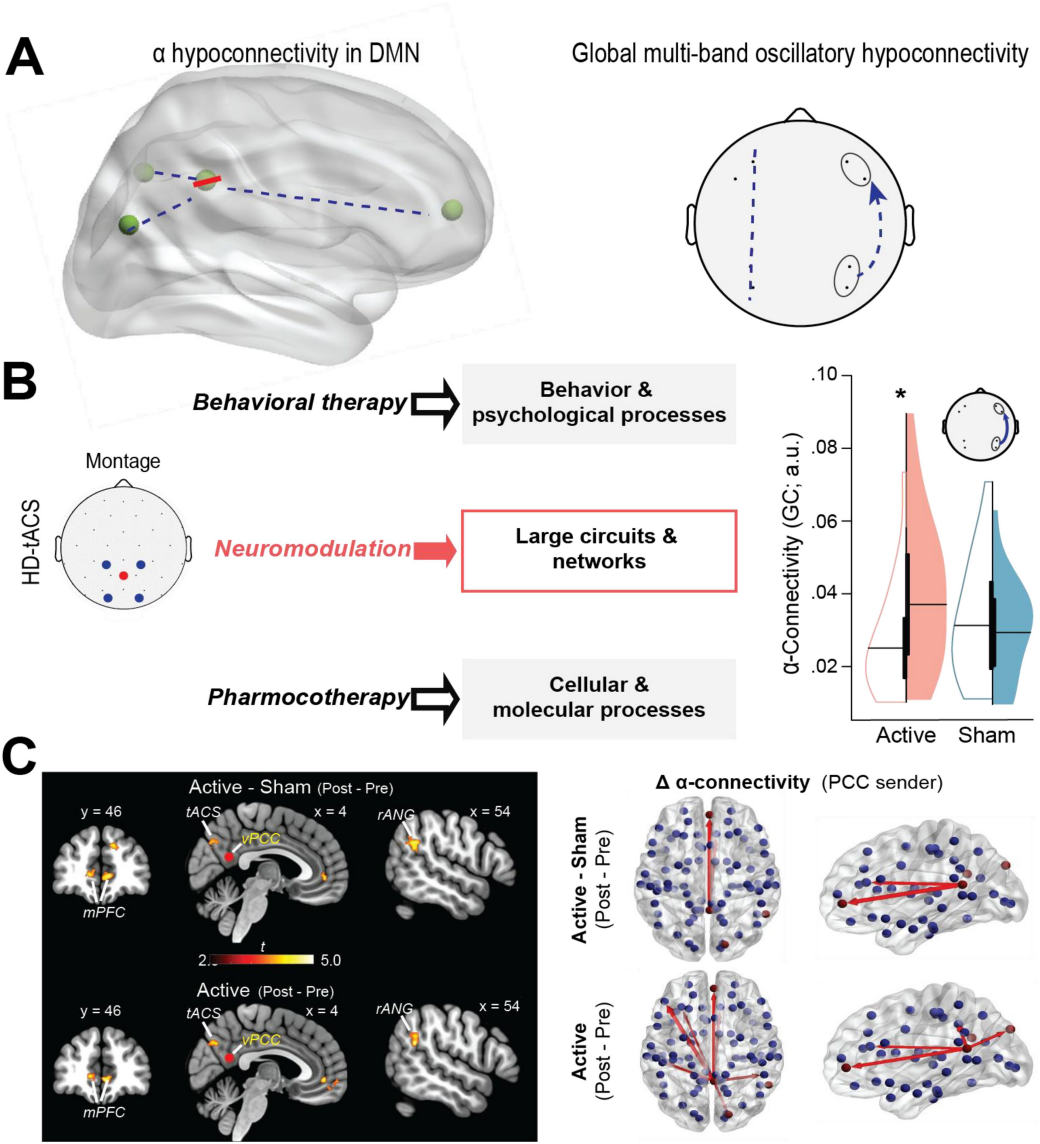
Interrogating and targeting oscillopathy-DMN dysfunction in AD via tACS. **(A)** AD is associated with reduced oscillatory (alpha and gamma) power and connectivity within the DMN and on the scalp. **(B)** Neuromodulation with tACS can directly target large-scale brain circuits and networks, bridging the longstanding gap between behavioral and pharmacological therapies, which primarily address psychological (Level V) and cellular/molecular (Level I/II) processes, respectively. High-definition tACS (HD-tACS) at the alpha frequency has been shown to enhance posterior-to-anterior alpha connectivity. **(C)** Critically, this stimulation augments DMN functional connectivity as measured by both fMRI and source-localized EEG, highlighting its potential to rectify DMN hypoconnectivity in AD. **B** and **C** adapted from Brown et al. (2025) and Clancy et al. (2022).

If oscillopathy contributes to DMN failure, then frequency-specific neuromodulation may restore network dynamics and improve cognition. This premise underlies a growing body of research employing transcranial alternating current stimulation (tACS) in Alzheimer’s disease (Figure 2).

### 4.1 Transcranial alternating current stimulation (tACS)

tACS delivers weak sinusoidal electric currents through the scalp to entrain endogenous oscillations, thereby enhancing or suppressing oscillatory activity within targeted frequency bands (Herrmann et al., 2013; Vosskuhl et al., 2018). By matching stimulation frequency to pathological rhythms, tACS can selectively modulate alpha slowing or gamma deficits. This frequency-specificity makes tACS particularly well suited for testing mechanistic hypotheses related to oscillopathy. For example, does enhancing alpha activity in the PCC restore appropriate DMN deactivation? Does augmenting gamma oscillations in MTL improve memory performance? Such targeted interventions enable causal interrogation of the proposed oscillopathy–DMN linkage, moving beyond correlational observations.

### 4.2 Mechanistically linking oscillopathy and DMN dysfunction

Recent studies have begun employing simultaneous EEG–fMRI and tACS paradigms to test whether targeted oscillatory modulation of specific oscillatory rhythms alters large-scale network dynamics. In healthy adults, combining simultaneous EEG-fMRI with alpha-frequency tACS over occipitoparietal region increased both EEG alpha power and (fMRI and EEG source-level) DMN connectivity, providing causal evidence that alpha oscillations contribute to DMN regulation (Clancy et al., 2022). Additionally, real-time coupling between spontaneous fluctuations in alpha power and DMN connectivity was shown to be enhanced by alpha-tACS (Ma et al., 2025), further supporting a direct oscillatory contribution to network-level dynamics. Although these studies were not conducted exclusively in AD populations, they establish a proof-of-concept that alpha oscillations can alter DMN dynamics. This framework motivates translational application of tACS to probe and potentially remediate oscillopathy-driven DMN dysfunction in AD (Figure 2).

### 4.3 Therapeutic applications in Alzheimer’s disease

Two complementary research streams target distinct oscillopathic phenotypes in AD. *Gamma-band (40 Hz) tACS* aims to restore local inhibitory circuit dynamics and has demonstrated improvement in memory and executive function, accompanied by increased gamma power, enhanced functional connectivity. Preliminary work also suggests potential downstream effects on tau pathology (Benussi et al., 2021; Dhaynaut et al., 2022). A meta-analysis of gamma-band tACS supports the therapeutic potential in AD, with evidence suggesting improvements in cognitive performance and acceptable safety across studies (Zhang et al., 2024).

*Alpha-band tACS* targets large-scale synchrony deficits by modulating intrinsic oscillatory activity linked to large-scale brain networks. Studies show that *α*-band tACS has been shown to enhance visuospatial and executive functions while increasing alpha-band activity in patients with dementia (Benussi et al., 2024). Also, α-tACS can modulate alpha-band connectivity and alter functional coupling within DMN (Clancy et al., 2022).

Together, these findings provide converging evidence that restoring pathological oscillatory dynamics—whether local gamma deficits or impaired alpha synchrony—may propagate upward to improve large-scale network (esp. DMN) function and cognition in AD.

### 4.4 Summary and limitations

Emerging tACS studies provide preliminary causal support for the proposed oscillopathy-DMN dysfunction framework. Frequency-specific modulation of alpha and gamma rhythms has been shown to alter oscillatory power, functional connectivity, and, in some cases, DMN dynamics, with associated cognitive improvements in AD and related populations. These findings suggest that pathological oscillations may contribute mechanistically to impaired large-scale network communication. By enabling targeted perturbation of specific frequency bands, tACS offers a translational bridge between mechanistic hypotheses and therapeutic interventions.

However, important limitations remain. Most tACS studies in AD are small, heterogeneous in design, and limited in durations. Moreover, changes in oscillatory measures do not necessarily indicate durable restoration of DMN integrity, as observed effects may reflect transient or compensatory processes. Furthermore, substantial interindividual variability in cortical anatomy, disease stage, and baseline oscillatory profiles complicates the interpretation. Larger, preregistered, multimodal trials integrating EEG and neuroimaging will be essential to determine whether oscillatory normalization via tACS yields sustained network restoration and clinically meaningful benefits across different stages of AD.

## 5 Implications in the era of AI

In the era of artificial intelligence (AI), the large-scale EEG data sets and their integration with multimodal biomarkers (e.g., fMRI, PET, structural MRI) create new opportunities for AI-driven diagnostics, prognostics, and personalized interventions strategies. The high-dimensional structure of oscillopathy—characterized by alpha slowing, gamma deficits, and distributed connectivity alteration—poses significant challenges for traditional one-size-fits-all analytical approaches. AI-driven frameworks provide scalable tools to model this complexity by identifying multivariate patterns within high-dimensional EEG data that relate to disease stage, network dysfunction and treatment response. Rather than isolating single-frequency metrics, machine learning approaches can integrate spectral, spatial, and connectivity features into predictive signatures that more accurately capture systems-level pathology.

Importantly, AI methods also address longstanding limitations of EEG biomarkers. Variability arising from methodological heterogeneity, pharmacological effects, and brain-state dependency has limited reproducibility. Machine learning and deep learning models are particularly well suited to extract stable, non-linear features across noisy and heterogenous datasets. When trained on sufficiently large and diverse cohorts, such models can distinguish trait-like oscillatory abnormalities from temporary state-related fluctuations. In this way, variability becomes less of a confound and more of an informative feature, supporting personalized monitoring and precision intervention.

### 5.1 Potential applications

#### 5.1.1 Diagnostic support

AI models trained on large-scale EEG datasets could enhance early detection and differential diagnosis of AD. Deep learning approaches such as convolutional neural networks (CNNs), recurrent neural networks (RNNs), and Transformers-based models can learn complex spatial and temporal features directly from raw EEG signals. These approaches may identify latent oscillatory signatures not captured by conventional spectral analyses, potentially improving classification performance when distinguishing AD from healthy controls and other dementias.

#### 5.1.2 Personalizing tACS

Baseline EEG features could inform individualized stimulation protocols. For example, alpha-frequency stimulation may be prioritized in patients exhibiting pronounced alpha slowing, whereas gamma-frequency stimulation may be more appropriate in those with local gamma deficits. Connectivity-informed approaches could further tailor stimulation targets based on specific network disconnection patterns. This “oscillopathy fingerprint” approach would move beyond one-size-fits-all stimulation paradigm toward precision neuromodulation strategies grounded in electrophysiological profiling.

#### 5.1.3 Closed-loop systems

Real-time EEG monitoring may enable adaptive stimulation that responds dynamically to fluctuations in brain state. Upon detecting oscillatory slowing or early signs of network decoupling, the system could deliver temporally targeted stimulation to stabilize network dynamics. Such closed-loop neuromodulation approaches are currently under active investigation and represent a promising direction for translating oscillopathy-based biomarkers into responsive therapeutic interventions (Jones et al., 2023; Zrenner et al., 2020).

## 6 Conclusion and future directions

This review proposes a conceptual framework linking oscillopathy and DMN dysfunction and highlighting their mechanistic and therapeutic targeting via tACS and AI. Within this model, oscillatory abnormalities act as a bridge between molecular pathology and DMN disruption. Disease progression may follow a hierarchical path from molecular and cellular changes, to local oscillatory abnormalities, impaired long-range synchrony, DMN failure and ultimately cognitive decline. tACS provides both a potential therapeutic strategy and a causal testing tool If frequency-specific stimulation restores DMN connectivity or improves cognition this would support a mechanistic role for oscillopathy in network dysfunction. At the same time, AI approaches allow high-dimensional characterization of oscillatory DMN patterns enabling patient stratification and the development of more personalized neuromodulation strategies. Although still in early stages, these approaches offer promising translational potential.

Owing to heterogeneity in analytic methods, disease stage, stimulation protocols, and cohort composition, some studies have reported null or inconsistent EEG and tACS findings. As this article presents a theoretical narrative synthesis rather than a systematic review, our aim has been to integrate converging evidence into a coherent working model. We acknowledge competing results and potential publication bias. The oscillopathy-DMN framework should therefore be considered a hypothesis that requires validation in larger, harmonized, and preregistered studies.

Future advances will depend on longitudinal, multimodal datasets that integrate EEG with imaging, fluid biomarkers, and cognitive assessment across the disease spectrum. While initiatives such as ADNI have begun incorporating EEG, current datasets remain limited. Although several oscillatory abnormalities are consistently reported in AD, their specificity for different clinical applications remains unclear. A key next step is to distinguish which EEG-based oscillatory markers are most useful for diagnosis (disease presence), prognosis (future decline), staging (disease severity), and treatment response (plasticity and modulation capacity). AI applied to large and heterogeneous datasets may help disentangle overlapping but distinct patterns and identify clinically meaningful application specific signatures.

## References

[ref1] Adaikkan C. Middleton S. J. Marco A. Pao P. C. Mathys H. Kim D. N. . (2019). Gamma entrainment binds higher-order brain regions and offers neuroprotection. Neuron 102, 929–943.e8. doi: 10.1016/j.neuron.2019.04.011 31076275 PMC6697125

[ref2] Akiki T. J. Averill C. L. Abdallah C. G. (2017). A network-based neurobiological model of PTSD: evidence from structural and functional neuroimaging studies. Curr. Psychiatry Rep. 19:81. doi: 10.1007/s11920-017-0840-4, 28924828 PMC5960989

[ref3] Alawode D. O. T. Fox N. C. Zetterberg H. Heslegrave A. J. (2022). Alzheimer's disease biomarkers revisited from the amyloid Cascade hypothesis standpoint. Front. Neurosci. 16:837390. doi: 10.3389/fnins.2022.837390, 35573283 PMC9091905

[ref4] Alzheimer's Association (2024). 2024 Alzheimer's disease facts and figures. Alzheimers Dement. 20, 3708–3821. doi: 10.1002/alz.13809, 38689398 PMC11095490

[ref5] Anticevic A. Cole M. W. Murray J. D. Corlett P. R. Wang X. J. Krystal J. H. (2012). The role of default network deactivation in cognition and disease. Trends Cogn. Sci. 16, 584–592. doi: 10.1016/j.tics.2012.10.008, 23142417 PMC3501603

[ref6] Babiloni C. Ferri R. Moretti D. V. Strambi A. Binetti G. Dal Forno G. . (2004). Abnormal fronto-parietal coupling of brain rhythms in mild Alzheimer's disease: a multicentric EEG study. Eur. J. Neurosci. 19, 2583–2590. doi: 10.1111/j.0953-816X.2004.03333.x, 15128412

[ref7] Babiloni C. Ferri R. Noce G. Lizio R. Lopez S. Soricelli A. . (2020). Resting-state electroencephalographic delta rhythms may reflect global cortical arousal in healthy old seniors and patients with Alzheimer's disease dementia. Int. J. Psychophysiol. 158, 259–270. doi: 10.1016/j.ijpsycho.2020.08.012, 33080295

[ref8] Babiloni C. Frisoni G. B. Pievani M. Vecchio F. Lizio R. Buttiglione M. . (2009). Hippocampal volume and cortical sources of EEG alpha rhythms in mild cognitive impairment and Alzheimer disease. NeuroImage 44, 123–135. doi: 10.1016/j.neuroimage.2008.08.005, 18805495

[ref9] Babiloni C. Lizio R. Del Percio C. Marzano N. Soricelli A. Salvatore E. . (2013). Cortical sources of resting state EEG rhythms are sensitive to the progression of early stage Alzheimer's disease. J. Alzheimer's Dis 34, 1015–1035. doi: 10.3233/JAD-121750, 23340039

[ref10] Badhwar A. Tam A. Dansereau C. Orban P. Hoffstaedter F. Bellec P. (2017). Resting-state network dysfunction in Alzheimer's disease: a systematic review and meta-analysis. Alzheimers Dement. 8, 73–85. doi: 10.1016/j.dadm.2017.03.007, 28560308 PMC5436069

[ref11] Bastos A. M. Schoffelen J.-M. (2016). A tutorial review of functional connectivity analysis methods and their interpretational pitfalls. Front. Syst. Neurosci. 9:175. doi: 10.3389/fnsys.2015.00175 26778976 PMC4705224

[ref12] Bennys K. Rondouin G. Vergnes C. Touchon J. (2001). Diagnostic value of quantitative EEG in Alzheimer's disease. Neurophysiol. Clin. 31, 153–160. doi: 10.1016/s0987-7053(01)00254-4, 11488226

[ref13] Benussi A. Cantoni V. Cotelli M. S. Cotelli M. Brattini C. Datta A. . (2021). Exposure to gamma tACS in Alzheimer's disease: a randomized, double-blind, sham-controlled, crossover, pilot study. Brain Stimul. 14, 531–540. doi: 10.1016/j.brs.2021.03.007, 33762220

[ref14] Benussi A. Cantoni V. Rivolta J. Zoppi N. Cotelli M. S. Bianchi M. . (2024). Alpha tACS improves cognition and modulates neurotransmission in dementia with Lewy bodies. Mov. Disord. 39, 1993–2003. doi: 10.1002/mds.29969, 39136447

[ref15] Benwell C. S. Y. Davila-Perez P. Fried P. J. Jones R. N. Travison T. G. Santarnecchi E. . (2020). EEG spectral power abnormalities and their relationship with cognitive dysfunction in patients with Alzheimer's disease and type 2 diabetes. Neurobiol. Aging 85, 83–95. doi: 10.1016/j.neurobiolaging.2019.10.004, 31727363 PMC6942171

[ref16] Blinowska K. J. Rakowski F. Kaminski M. De Vico Fallani F. Del Percio C. Lizio R. . (2017). Functional and effective brain connectivity for discrimination between Alzheimer's patients and healthy individuals: a study on resting state EEG rhythms. Clin. Neurophysiol. 128, 667–680. doi: 10.1016/j.clinph.2016.10.002, 27836429

[ref17] Braak H. Braak E. (1991). Neuropathological stageing of Alzheimer-related changes. Acta Neuropathol. 82, 239–259. doi: 10.1007/BF00308809, 1759558

[ref18] Braak H. Del Tredici K. (2014). Are cases with tau pathology occurring in the absence of Abeta deposits part of the AD-related pathological process? Acta Neuropathol. 128, 767–772. doi: 10.1007/s00401-014-1356-1, 25359108

[ref19] Braak H. Del Tredici K. (2015). The preclinical phase of the pathological process underlying sporadic Alzheimer's disease. Brain 138, 2814–2833. doi: 10.1093/brain/awv236, 26283673

[ref20] Briels C. T. Schoonhoven D. N. Stam C. J. de Waal H. Scheltens P. Gouw A. A. (2020a). Reproducibility of EEG functional connectivity in Alzheimer's disease. Alzheimer's Res. Ther. 12:68. doi: 10.1186/s13195-020-00632-3, 32493476 PMC7271479

[ref21] Briels C. T. Stam C. J. Scheltens P. Bruins S. Lues I. Gouw A. A. (2020b). In pursuit of a sensitive EEG functional connectivity outcome measure for clinical trials in Alzheimer's disease. Clin. Neurophysiol. 131, 88–95. doi: 10.1016/j.clinph.2019.09.014, 31759193

[ref22] Brier M. R. Thomas J. B. Ances B. M. (2014). Network dysfunction in Alzheimer's disease: refining the disconnection hypothesis. Brain Connect. 4, 299–311. doi: 10.1089/brain.2014.0236, 24796856 PMC4064730

[ref23] Brown J. A. Clancy K. J. Li W. (2025). “Neuromodulation of fear and anxiety circuits,” in New Discoveries in the Brain Sciences of Fear and Anxiety - From Basic to Clinical Neuroscience, eds. Blackford J. U. Milad M. R. (Cham: Springer Nature Switzerland), 407–431. 10.1007/7854_2024_573 PMC13596488 40067414

[ref24] Bruns A. Eckhorn R. (2004). Task-related coupling from high- to low-frequency signals among visual cortical areas in human subdural recordings. Int. J. Psychophysiol. 51, 97–116. doi: 10.1016/j.ijpsycho.2003.07.001, 14693360

[ref25] Buckner R. L. Snyder A. Z. Shannon B. J. LaRossa G. Sachs R. Fotenos A. F. . (2005). Molecular, structural, and functional characterization of Alzheimer's disease: evidence for a relationship between default activity, amyloid, and memory. J. Neurosci. 25, 7709–7717. doi: 10.1523/JNEUROSCI.2177-05.2005, 16120771 PMC6725245

[ref26] Buzsaki G. Draguhn A. (2004). Neuronal oscillations in cortical networks. Science 304, 1926–1929. doi: 10.1126/science.1099745, 15218136

[ref27] Cardin J. A. Carlen M. Meletis K. Knoblich U. Zhang F. Deisseroth K. . (2009). Driving fast-spiking cells induces gamma rhythm and controls sensory responses. Nature 459, 663–667. doi: 10.1038/nature08002, 19396156 PMC3655711

[ref28] Cassani R. Estarellas M. San-Martin R. Fraga F. J. Falk T. H. (2018). Systematic review on resting-state EEG for Alzheimer's disease diagnosis and progression assessment. Dis. Markers 2018:5174815. doi: 10.1155/2018/5174815, 30405860 PMC6200063

[ref29] Casula E. P. Pellicciari M. C. Bonni S. Borghi I. Maiella M. Assogna M. . (2022). Decreased frontal gamma activity in Alzheimer disease patients. Ann. Neurol. 92, 464–475. doi: 10.1002/ana.26444, 35713198 PMC9543336

[ref30] Cecchetti G. Agosta F. Basaia S. Cividini C. Cursi M. Santangelo R. . (2021). Resting-state electroencephalographic biomarkers of Alzheimer's disease. Neuroimage Clin. 31:102711. doi: 10.1016/j.nicl.2021.102711, 34098525 PMC8185302

[ref31] Clancy K. J. Andrzejewski J. A. You Y. Rosenberg J. T. Ding M. Li W. (2022). Transcranial stimulation of alpha oscillations up-regulates the default mode network. Proc. Natl. Acad. Sci. USA 119:e2110868119. doi: 10.1073/pnas.2110868119, 34969856 PMC8740757

[ref32] Clark C. M. Pontecorvo M. J. Beach T. G. Bedell B. J. Coleman R. E. Doraiswamy P. M. . (2012). Cerebral PET with florbetapir compared with neuropathology at autopsy for detection of neuritic amyloid-beta plaques: a prospective cohort study. Lancet Neurol. 11, 669–678. doi: 10.1016/S1474-4422(12)70142-4 22749065

[ref33] Coito A. Michel C. M. Vulliemoz S. Plomp G. (2019). Directed functional connections underlying spontaneous brain activity. Hum. Brain Mapp. 40, 879–888. doi: 10.1002/hbm.24418, 30367722 PMC6865461

[ref34] Dauwan M. van Dellen E. van Boxtel L. van Straaten E. C. W. de Waal H. Lemstra A. W. . (2016). EEG-directed connectivity from posterior brain regions is decreased in dementia with Lewy bodies: a comparison with Alzheimer's disease and controls. Neurobiol. Aging 41, 122–129. doi: 10.1016/j.neurobiolaging.2016.02.017, 27103525

[ref35] Delbeuck X. Van der Linden M. Collette F. (2003). Alzheimer's disease as a disconnection syndrome? Neuropsychol. Rev. 13, 79–92. doi: 10.1023/a:1023832305702, 12887040

[ref36] Dennis E. L. Thompson P. M. (2014). Functional brain connectivity using fMRI in aging and Alzheimer's disease. Neuropsychol. Rev. 24, 49–62. doi: 10.1007/s11065-014-9249-6, 24562737 PMC4109887

[ref37] Dhaynaut M. Sprugnoli G. Cappon D. Macone J. Sanchez J. S. Normandin M. D. . (2022). Impact of 40 Hz transcranial alternating current stimulation on cerebral tau burden in patients with Alzheimer's disease: a case series. J. Alzheimer's Dis 85, 1667–1676. doi: 10.3233/JAD-215072, 34958021 PMC9023125

[ref38] Engel A. K. Fries P. (2010). Beta-band oscillations—signalling the status quo? Curr. Opin. Neurobiol. 20, 156–165. doi: 10.1016/j.conb.2010.02.015, 20359884

[ref39] Engel A. K. Fries P. Singer W. (2001). Dynamic predictions: oscillations and synchrony in top-down processing. Nat. Rev. Neurosci. 2, 704–716. doi: 10.1038/35094565, 11584308

[ref40] Engels M. M. A. Yu M. Stam C. J. Gouw A. A. van der Flier W. M. Scheltens P. . (2017). Directional information flow in patients with Alzheimer's disease. A source-space resting-state MEG study. Neuroimage Clin. 15, 673–681. doi: 10.1016/j.nicl.2017.06.025, 28702344 PMC5486371

[ref41] Etkin A. Mathalon D. H. (2024). Bringing imaging biomarkers into clinical reality in psychiatry. JAMA Psychiatry 81, 1142–1147. doi: 10.1001/jamapsychiatry.2024.2553, 39230917

[ref42] Fischer M. H. F. Zibrandtsen I. C. Hogh P. Musaeus C. S. (2023). Systematic review of EEG coherence in Alzheimer's disease. J. Alzheimer's Dis 91, 1261–1272. doi: 10.3233/JAD-220508, 36641665

[ref43] Fox M. D. Snyder A. Z. Vincent J. L. Corbetta M. Van Essen D. C. Raichle M. E. (2005). The human brain is intrinsically organized into dynamic, anticorrelated functional networks. Proc. Natl. Acad. Sci. USA 102, 9673–9678. doi: 10.1073/pnas.0504136102, 15976020 PMC1157105

[ref44] Foxe J. J. Snyder A. C. (2011). The role of alpha-band brain oscillations as a sensory suppression mechanism during selective attention. Front. Psychol. 2:154. doi: 10.3389/fpsyg.2011.00154, 21779269 PMC3132683

[ref45] Fries P. (2005). A mechanism for cognitive dynamics: neuronal communication through neuronal coherence. Trends Cogn. Sci. 9, 474–480. doi: 10.1016/j.tics.2005.08.011, 16150631

[ref46] Gaubert S. Raimondo F. Houot M. Corsi M. C. Naccache L. Diego Sitt J. . (2019). EEG evidence of compensatory mechanisms in preclinical Alzheimer's disease. Brain 142, 2096–2112. doi: 10.1093/brain/awz150, 31211359

[ref47] Goodman M. S. Kumar S. Zomorrodi R. Ghazala Z. Cheam A. S. M. Barr M. S. . (2018). Theta-gamma coupling and working memory in Alzheimer's dementia and mild cognitive impairment. Front. Aging Neurosci. 10:101. doi: 10.3389/fnagi.2018.00101, 29713274 PMC5911490

[ref48] Greicius M. D. Flores B. H. Menon V. Glover G. H. Solvason H. B. Kenna H. . (2007). Resting-state functional connectivity in major depression: abnormally increased contributions from subgenual cingulate cortex and thalamus. Biol. Psychiatry 62, 429–437. doi: 10.1016/j.biopsych.2006.09.020, 17210143 PMC2001244

[ref49] Greicius M. D. Srivastava G. Reiss A. L. Menon V. (2004). Default-mode network activity distinguishes Alzheimer's disease from healthy aging: evidence from functional MRI. Proc. Natl. Acad. Sci. USA 101, 4637–4642. doi: 10.1073/pnas.0308627101, 15070770 PMC384799

[ref50] Hata M. Kazui H. Tanaka T. Ishii R. Canuet L. Pascual-Marqui R. D. . (2016). Functional connectivity assessed by resting state EEG correlates with cognitive decline of Alzheimer's disease—an eLORETA study. Clin. Neurophysiol. 127, 1269–1278. doi: 10.1016/j.clinph.2015.10.030, 26541308

[ref51] Heidebrink J. L. Paulson H. L. (2024). Lessons learned from approval of aducanumab for Alzheimer's disease. Annu. Rev. Med. 75, 99–111. doi: 10.1146/annurev-med-051022-043645, 38285515 PMC10926277

[ref52] Herrmann C. S. Rach S. Neuling T. Struber D. (2013). Transcranial alternating current stimulation: a review of the underlying mechanisms and modulation of cognitive processes. Front. Hum. Neurosci. 7:279. doi: 10.3389/fnhum.2013.00279, 23785325 PMC3682121

[ref53] Hillebrand A. Tewarie P. van Dellen E. Yu M. Carbo E. W. Douw L. . (2016). Direction of information flow in large-scale resting-state networks is frequency-dependent. Proc. Natl. Acad. Sci. USA 113, 3867–3872. doi: 10.1073/pnas.1515657113, 27001844 PMC4833227

[ref54] Huang C. Wahlund L. Dierks T. Julin P. Winblad B. Jelic V. (2000). Discrimination of Alzheimer's disease and mild cognitive impairment by equivalent EEG sources: a cross-sectional and longitudinal study. Clin. Neurophysiol. 111, 1961–1967. doi: 10.1016/s1388-2457(00)00454-5, 11068230

[ref55] Hyafil A. Giraud A. L. Fontolan L. Gutkin B. (2015). Neural cross-frequency coupling: connecting architectures, mechanisms, and functions. Trends Neurosci. 38, 725–740. doi: 10.1016/j.tins.2015.09.001, 26549886

[ref56] Jann K. Dierks T. Boesch C. Kottlow M. Strik W. Koenig T. (2009). BOLD correlates of EEG alpha phase-locking and the fMRI default mode network. NeuroImage 45, 903–916. doi: 10.1016/j.neuroimage.2009.01.001, 19280706

[ref57] Jelic V. Johansson S. E. Almkvist O. Shigeta M. Julin P. Nordberg A. . (2000). Quantitative electroencephalography in mild cognitive impairment: longitudinal changes and possible prediction of Alzheimer's disease. Neurobiol. Aging 21, 533–540. doi: 10.1016/s0197-4580(00)00153-6, 10924766

[ref58] Jensen O. Mazaheri A. (2010). Shaping functional architecture by oscillatory alpha activity: gating by inhibition. Front. Hum. Neurosci. 4:186. doi: 10.3389/fnhum.2010.00186, 21119777 PMC2990626

[ref59] Jiang Y. Neal J. Sompol P. Yener G. Arakaki X. Norris C. M. . (2024). Parallel electrophysiological abnormalities due to COVID-19 infection and to Alzheimer's disease and related dementia. Alzheimers Dement. 20, 7296–7319. doi: 10.1002/alz.14089, 39206795 PMC11485397

[ref60] Jones K. T. Gallen C. L. Ostrand A. E. Rojas J. C. Wais P. Rini J. . (2023). Gamma neuromodulation improves episodic memory and its associated network in amnestic mild cognitive impairment: a pilot study. Neurobiol. Aging 129, 72–88. doi: 10.1016/j.neurobiolaging.2023.04.005, 37276822 PMC10583532

[ref61] Jovicich J. Babiloni C. Ferrari C. Marizzoni M. Moretti D. V. Del Percio C. . (2019). Two-year longitudinal monitoring of amnestic mild cognitive impairment patients with prodromal Alzheimer's disease using topographical biomarkers derived from functional magnetic resonance imaging and electroencephalographic activity. J. Alzheimer's Dis 69, 15–35. doi: 10.3233/JAD-180158, 30400088

[ref62] Juan-Cruz C. Gómez C. Poza J. Fernández A. Hornero R. (2016) Analysis of magnetoencephalography signals from Alzheimer's disease patients using granger causality. Paper presented at the 2016 38th Annual International Conference of the IEEE Engineering in Medicine and Biology Society (EMBC) 10.1109/EMBC.2016.7590804 28268430

[ref63] Karch C. M. Goate A. M. (2015). Alzheimer's disease risk genes and mechanisms of disease pathogenesis. Biol. Psychiatry 77, 43–51. doi: 10.1016/j.biopsych.2014.05.006, 24951455 PMC4234692

[ref64] Kirschner A. Kam J. W. Handy T. C. Ward L. M. (2012). Differential synchronization in default and task-specific networks of the human brain. Front. Hum. Neurosci. 6:139. doi: 10.3389/fnhum.2012.00139, 22661936 PMC3356872

[ref65] Klimesch W. (1999). EEG alpha and theta oscillations reflect cognitive and memory performance: a review and analysis. Brain Res. Brain Res. Rev. 29, 169–195. doi: 10.1016/s0165-0173(98)00056-3, 10209231

[ref66] Klimesch W. Sauseng P. Hanslmayr S. (2007). EEG alpha oscillations: the inhibition-timing hypothesis. Brain Res. Rev. 53, 63–88. doi: 10.1016/j.brainresrev.2006.06.003, 16887192

[ref67] Koenig T. Prichep L. Dierks T. Hubl D. Wahlund L. O. John E. R. . (2005). Decreased EEG synchronization in Alzheimer's disease and mild cognitive impairment. Neurobiol. Aging 26, 165–171. doi: 10.1016/j.neurobiolaging.2004.03.008, 15582746

[ref68] Koster M. Finger H. Graetz S. Kater M. Gruber T. (2018). Theta-gamma coupling binds visual perceptual features in an associative memory task. Sci. Rep. 8:17688. doi: 10.1038/s41598-018-35812-7, 30523336 PMC6283876

[ref69] Kramberger M. G. Kareholt I. Andersson T. Winblad B. Eriksdotter M. Jelic V. (2013). Association between EEG abnormalities and CSF biomarkers in a memory clinic cohort. Dement. Geriatr. Cogn. Disord. 36, 319–328. doi: 10.1159/000351677, 24022277

[ref70] Kwong K. K. Belliveau J. W. Chesler D. A. Goldberg I. E. Weisskoff R. M. Poncelet B. P. . (1992). Dynamic magnetic resonance imaging of human brain activity during primary sensory stimulation. Proc. Natl. Acad. Sci. USA 89, 5675–5679. doi: 10.1073/pnas.89.12.5675, 1608978 PMC49355

[ref71] Lachaux J. P. Rodriguez E. Martinerie J. Varela F. J. (1999). Measuring phase synchrony in brain signals. Hum. Brain Mapp. 8, 194–208. doi: 10.1002/(sici)1097-0193(1999)8:4<194::aid-hbm4>3.0.co;2-c, 10619414 PMC6873296

[ref72] Leuzy A. Smith R. Cullen N. C. Strandberg O. Vogel J. W. Binette A. P. . (2022). Biomarker-based prediction of longitudinal tau positron emission tomography in Alzheimer disease. JAMA Neurol. 79, 149–158. doi: 10.1001/jamaneurol.2021.4654, 34928318 PMC8689441

[ref73] Li Z. Shue F. Zhao N. Shinohara M. Bu G. (2020). APOE2: protective mechanism and therapeutic implications for Alzheimer's disease. Mol. Neurodegener. 15:63. doi: 10.1186/s13024-020-00413-4, 33148290 PMC7640652

[ref74] Llinas R. R. Ribary U. Jeanmonod D. Kronberg E. Mitra P. P. (1999). Thalamocortical dysrhythmia: a neurological and neuropsychiatric syndrome characterized by magnetoencephalography. Proc. Natl. Acad. Sci. USA 96, 15222–15227. doi: 10.1073/pnas.96.26.15222, 10611366 PMC24801

[ref75] Love S. Miners J. S. (2016). Cerebral hypoperfusion and the energy deficit in Alzheimer's disease. Brain Pathol. 26, 607–617. doi: 10.1111/bpa.12401, 27327656 PMC8028913

[ref76] Lustig C. Snyder A. Z. Bhakta M. O'Brien K. C. McAvoy M. Raichle M. E. . (2003). Functional deactivations: change with age and dementia of the Alzheimer type. Proc. Natl. Acad. Sci. USA 100, 14504–14509. doi: 10.1073/pnas.2235925100, 14608034 PMC283621

[ref77] Lutz A. Greischar L. L. Rawlings N. B. Ricard M. Davidson R. J. (2004). Long-term meditators self-induce high-amplitude gamma synchrony during mental practice. Proc. Natl. Acad. Sci. USA 101, 16369–16373. doi: 10.1073/pnas.0407401101, 15534199 PMC526201

[ref78] Ma Y. Brown J. A. Chen C. Ding M. Wu W. Li W. (2025). Alpha-frequency stimulation enhances synchronization of alpha oscillations with default mode network connectivity. eNeuro 12:ENEURO.0449-0424.2025. doi: 10.1523/eneuro.0449-24.2025, 40068873 PMC11927933

[ref79] Ma C. C. Liu A. J. Liu A. H. Zhou X. Y. Zhou S. N. (2014). Electroencephalogram global field synchronization analysis: a new method for assessing the progress of cognitive decline in Alzheimer's disease. Clin. EEG Neurosci. 45, 98–103. doi: 10.1177/1550059413489669, 23986293

[ref80] Margulies D. S. Ghosh S. S. Goulas A. Falkiewicz M. Huntenburg J. M. Langs G. . (2016). Situating the default-mode network along a principal gradient of macroscale cortical organization. Proc. Natl. Acad. Sci. USA 113, 12574–12579. doi: 10.1073/pnas.1608282113, 27791099 PMC5098630

[ref81] Martorell A. J. Paulson A. L. Suk H. J. Abdurrob F. Drummond G. T. Guan W. . (2019). Multi-sensory gamma stimulation ameliorates Alzheimer's-associated pathology and improves cognition. Cell 177, 256–271 e222. doi: 10.1016/j.cell.2019.02.014 30879788 PMC6774262

[ref82] Mattsson-Carlgren N. Grinberg L. T. Boxer A. Ossenkoppele R. Jonsson M. Seeley W. . (2022). Cerebrospinal fluid biomarkers in autopsy-confirmed Alzheimer disease and frontotemporal lobar degeneration. Neurology 98, e1137–e1150. doi: 10.1212/WNL.0000000000200040, 35173015 PMC8935438

[ref83] McMackin R. Bede P. Pender N. Hardiman O. Nasseroleslami B. (2019). Neurophysiological markers of network dysfunction in neurodegenerative diseases. Neuroimage Clin. 22:101706. doi: 10.1016/j.nicl.2019.101706, 30738372 PMC6370863

[ref84] Montagne A. Nation D. A. Sagare A. P. Barisano G. Sweeney M. D. Chakhoyan A. . (2020). APOE4 leads to blood-brain barrier dysfunction predicting cognitive decline. Nature 581, 71–76. doi: 10.1038/s41586-020-2247-3, 32376954 PMC7250000

[ref85] Moretti D. V. Babiloni C. Binetti G. Cassetta E. Dal Forno G. Ferreric F. . (2004). Individual analysis of EEG frequency and band power in mild Alzheimer's disease. Clin. Neurophysiol. 115, 299–308. doi: 10.1016/s1388-2457(03)00345-6, 14744569

[ref86] Musaeus C. S. Engedal K. Hogh P. Jelic V. Morup M. Naik M. . (2018). EEG Theta power is an early marker of cognitive decline in dementia due to Alzheimer's disease. J. Alzheimer's Dis 64, 1359–1371. doi: 10.3233/JAD-180300, 29991135

[ref87] Musiek E. S. Bhimasani M. Zangrilli M. A. Morris J. C. Holtzman D. M. Ju Y. S. (2018). Circadian rest-activity pattern changes in aging and preclinical Alzheimer disease. JAMA Neurol. 75, 582–590. doi: 10.1001/jamaneurol.2017.4719, 29379963 PMC5885197

[ref88] Musiek E. S. Holtzman D. M. (2015). Three dimensions of the amyloid hypothesis: time, space and 'wingmen'. Nat. Neurosci. 18, 800–806. doi: 10.1038/nn.4018, 26007213 PMC4445458

[ref89] Ozbek Y. Fide E. Yener G. G. (2021). Resting-state EEG alpha/theta power ratio discriminates early-onset Alzheimer's disease from healthy controls. Clin. Neurophysiol. 132, 2019–2031. doi: 10.1016/j.clinph.2021.05.012, 34284236

[ref90] Palmqvist S. Scholl M. Strandberg O. Mattsson N. Stomrud E. Zetterberg H. . (2017). Earliest accumulation of β-amyloid occurs within the default-mode network and concurrently affects brain connectivity. Nat. Commun. 8:1214. doi: 10.1038/s41467-017-01150-x, 29089479 PMC5663717

[ref91] Palop J. J. Mucke L. (2016). Network abnormalities and interneuron dysfunction in Alzheimer disease. Nat. Rev. Neurosci. 17, 777–792. doi: 10.1038/nrn.2016.141, 27829687 PMC8162106

[ref92] Palva S. Palva J. M. (2007). New vistas for alpha-frequency band oscillations. Trends Neurosci. 30, 150–158. doi: 10.1016/j.tins.2007.02.001, 17307258

[ref93] Park Y. M. Che H. J. Im C. H. Jung H. T. Bae S. M. Lee S. H. (2008). Decreased EEG synchronization and its correlation with symptom severity in Alzheimer's disease. Neurosci. Res. 62, 112–117. doi: 10.1016/j.neures.2008.06.009, 18672010

[ref94] Pascual-Marqui R. D. (2007). Discrete, 3D distributed, linear imaging methods of electric neuronal activity. Part 1: exact, zero error localization. arXiv. doi: 10.48550/arXiv.0710.3341

[ref95] Pascual-Marqui R. D. Michel C. M. Lehmann D. (1994). Low resolution electromagnetic tomography: a new method for localizing electrical activity in the brain. Int. J. Psychophysiol. 18, 49–65. doi: 10.1016/0167-8760(84)90014-x, 7876038

[ref96] Pathak A. Sharma V. Roy D. Banerjee A. (2022). Biophysical mechanism underlying compensatory preservation of neural synchrony over the adult lifespan. Commun. Biol. 5:567. doi: 10.1038/s42003-022-03489-4, 35681107 PMC9184644

[ref97] Qin Y. Menara T. Bassett D. S. Pasqualetti F. (2021). Phase-amplitude coupling in neuronal oscillator networks. Phys. Rev. Res. 3:023218. doi: 10.1103/PhysRevResearch.3.023218

[ref98] Raichle M. E. (2015). The brain's default mode network. Annu. Rev. Neurosci. 38, 433–447. doi: 10.1146/annurev-neuro-071013-014030, 25938726

[ref99] Raichle M. E. MacLeod A. M. Snyder A. Z. Powers W. J. Gusnard D. A. Shulman G. L. (2001). A default mode of brain function. Proc. Natl. Acad. Sci. 98, 676–682. doi: 10.1073/pnas.98.2.676, 11209064 PMC14647

[ref100] Raichle M. E. Snyder A. Z. (2007). A default mode of brain function: a brief history of an evolving idea. NeuroImage 37:1083-1090; discussion 1097-1089. doi: 10.1016/j.neuroimage.2007.02.041, 17719799

[ref101] Roh J. H. Park M. H. Ko D. Park K. W. Lee D. H. Han C. . (2011). Region and frequency specific changes of spectral power in Alzheimer's disease and mild cognitive impairment. Clin. Neurophysiol. 122, 2169–2176. doi: 10.1016/j.clinph.2011.03.023, 21715226

[ref102] Samogin J. Liu Q. Marino M. Wenderoth N. Mantini D. (2019). Shared and connection-specific intrinsic interactions in the default mode network. NeuroImage 200, 474–481. doi: 10.1016/j.neuroimage.2019.07.007, 31280013

[ref103] Scheeringa R. Petersson K. M. Kleinschmidt A. Jensen O. Bastiaansen M. C. (2012). EEG alpha power modulation of fMRI resting-state connectivity. Brain Connect. 2, 254–264. doi: 10.1089/brain.2012.0088, 22938826 PMC3621304

[ref104] Sheline Y. I. Raichle M. E. (2013). Resting state functional connectivity in preclinical Alzheimer's disease. Biol. Psychiatry 74, 340–347. doi: 10.1016/j.biopsych.2012.11.028, 23290495 PMC3537262

[ref105] Smailovic U. Koenig T. Kareholt I. Andersson T. Kramberger M. G. Winblad B. . (2018). Quantitative EEG power and synchronization correlate with Alzheimer's disease CSF biomarkers. Neurobiol. Aging 63, 88–95. doi: 10.1016/j.neurobiolaging.2017.11.005, 29245058

[ref106] Sohal V. S. Zhang F. Yizhar O. Deisseroth K. (2009). Parvalbumin neurons and gamma rhythms enhance cortical circuit performance. Nature 459, 698–702. doi: 10.1038/nature07991, 19396159 PMC3969859

[ref107] Sorg C. Riedl V. Muhlau M. Calhoun V. D. Eichele T. Laer L. . (2007). Selective changes of resting-state networks in individuals at risk for Alzheimer's disease. Proc. Natl. Acad. Sci. USA 104, 18760–18765. doi: 10.1073/pnas.0708803104, 18003904 PMC2141850

[ref108] Szczepanski S. M. Crone N. E. Kuperman R. A. Auguste K. I. Parvizi J. Knight R. T. (2014). Dynamic changes in phase-amplitude coupling facilitate spatial attention control in fronto-parietal cortex. PLoS Biol. 12:e1001936. doi: 10.1371/journal.pbio.1001936, 25157678 PMC4144794

[ref109] Tagliazucchi E. von Wegner F. Morzelewski A. Brodbeck V. Laufs H. (2012). Dynamic BOLD functional connectivity in humans and its electrophysiological correlates. Front. Hum. Neurosci. 6:339. doi: 10.3389/fnhum.2012.00339, 23293596 PMC3531919

[ref110] Tang W. Liu H. Douw L. Kramer M. A. Eden U. T. Hamalainen M. S. . (2017). Dynamic connectivity modulates local activity in the core regions of the default-mode network. Proc. Natl. Acad. Sci. USA 114, 9713–9718. doi: 10.1073/pnas.1702027114, 28827337 PMC5594646

[ref111] Tarantini S. Tran C. H. T. Gordon G. R. Ungvari Z. Csiszar A. (2017). Impaired neurovascular coupling in aging and Alzheimer's disease: contribution of astrocyte dysfunction and endothelial impairment to cognitive decline. Exp. Gerontol. 94, 52–58. doi: 10.1016/j.exger.2016.11.004, 27845201 PMC5429210

[ref113] Uhlhaas P. J. Pipa G. Lima B. Melloni L. Neuenschwander S. Nikolic D. . (2009). Neural synchrony in cortical networks: history, concept and current status. Front. Integr. Neurosci. 3:17. doi: 10.3389/neuro.07.017.2009, 19668703 PMC2723047

[ref114] van der Hiele K. Vein A. A. Reijntjes R. H. Westendorp R. G. Bollen E. L. van Buchem M. A. . (2007). EEG correlates in the spectrum of cognitive decline. Clin. Neurophysiol. 118, 1931–1939. doi: 10.1016/j.clinph.2007.05.070, 17604688

[ref115] van Deursen J. A. Vuurman E. F. Verhey F. R. van Kranen-Mastenbroek V. H. Riedel W. J. (2008). Increased EEG gamma band activity in Alzheimer's disease and mild cognitive impairment. J. Neural Transm. (Vienna) 115, 1301–1311. doi: 10.1007/s00702-008-0083-y, 18607528 PMC2525849

[ref116] Varela F. Lachaux J. P. Rodriguez E. Martinerie J. (2001). The brainweb: phase synchronization and large-scale integration. Nat. Rev. Neurosci. 2, 229–239. doi: 10.1038/35067550, 11283746

[ref112] Vespa J. Medina L. Armstrong D. M. (2020). Demographic Turning Points for the United States: Population Projections for 2020 to 2060. Current Population Reports, 25–1144. Washington, DC: U.S. Census Bureau. Available online at: https://www.census.gov/content/dam/Census/library/publications/2020/demo/p25-1144.pdf

[ref117] Verret L. Mann E. O. Hang G. B. Barth A. M. Cobos I. Ho K. . (2012). Inhibitory interneuron deficit links altered network activity and cognitive dysfunction in Alzheimer model. Cell 149, 708–721. doi: 10.1016/j.cell.2012.02.046, 22541439 PMC3375906

[ref118] Vosskuhl J. Struber D. Herrmann C. S. (2018). Non-invasive brain stimulation: a paradigm shift in understanding brain oscillations. Front. Hum. Neurosci. 12:211. doi: 10.3389/fnhum.2018.00211, 29887799 PMC5980979

[ref119] Wang J. Zhao J. Chen X. Yin B. Li X. Xie P. (2025). Alzheimer’s disease diagnosis using rhythmic power changes and phase differences: a low-density EEG study. Front. Aging Neurosci. 16:1485132. doi: 10.3389/fnagi.2024.1485132, 39897456 PMC11782140

[ref120] Whitfield-Gabrieli S. Thermenos H. W. Milanovic S. Tsuang M. T. Faraone S. V. McCarley R. W. . (2009). Hyperactivity and hyperconnectivity of the default network in schizophrenia and in first-degree relatives of persons with schizophrenia. Proc. Natl. Acad. Sci. USA 106, 1279–1284. doi: 10.1073/pnas.0809141106, 19164577 PMC2633557

[ref121] Xiong M. Jiang H. Serrano J. R. Gonzales E. R. Wang C. Gratuze M. . (2021). APOE immunotherapy reduces cerebral amyloid angiopathy and amyloid plaques while improving cerebrovascular function. Sci. Transl. Med. 13:eabd7522. doi: 10.1126/scitranslmed.abd7522, 33597265 PMC8128342

[ref122] Zhang X. Lv R. Sun Y. Liu T. C. (2024). The safety and effectiveness of 40 Hz gamma-tACS in Alzheimer's disease: a meta-analysis. J. Alzheimer's Dis 102, 295–307. doi: 10.1177/13872877241289397, 39573866

[ref123] Zhang C. Yu X. Yang Y. Xu L. (2014). Phase synchronization and spectral coherence analysis of EEG activity during mental fatigue. Clin. EEG Neurosci. 45, 249–256. doi: 10.1177/1550059413503961, 24590874

[ref124] Zheng X. Wang B. Liu H. Wu W. Sun J. Fang W. . (2023). Diagnosis of Alzheimer's disease via resting-state EEG: integration of spectrum, complexity, and synchronization signal features. Front. Aging Neurosci. 15:1288295. doi: 10.3389/fnagi.2023.1288295, 38020761 PMC10661409

[ref125] Zrenner B. Zrenner C. Gordon P. C. Belardinelli P. McDermott E. J. Soekadar S. R. . (2020). Brain oscillation-synchronized stimulation of the left dorsolateral prefrontal cortex in depression using real-time EEG-triggered TMS. Brain Stimul. 13, 197–205. doi: 10.1016/j.brs.2019.10.007, 31631058

